# A New Disk

**Published:** 1883-05

**Authors:** 


### A New Disk.

We have, for a few weeks, been using albuminum disks, devised and made by Dr. E. B. Call, of Peoria, Ill. They are used with corrundum flower. They cut and polish about as well as the diamond disk—much better than some diamond disks we have had. They are cheaper, they are always ready for work without special charging, except moistening and sprinkling on the flour; they are thinner, which in many cases is desirable, they will cut upon the edge, which the diamond disk, after a little use at least, will not. They will not wholly supersede the good diamond disk, but will the baser sort that have sometimes been in the market.

Try them, but as in the use of all disks, with great care.

